# Editorial: Cutting Edge Robotic Techniques: From Performance to Teaching to Telemedicine

**DOI:** 10.3389/fsurg.2021.703498

**Published:** 2021-08-04

**Authors:** Robert J. Cerfolio

**Affiliations:** Department of Cardiothoracic Surgery, New York University Langone Health, New York, NY, United States

**Keywords:** robotic lung surgery, efficiency quality index, telemedicine, quality outcomes, education

It is my honor to write a brief editorial for this exciting section, “Cutting Edge Robotic Techniques: From Performance to Teaching to Telemedicine.” I want to thank the authors for the hard work and sedulousness to create this section and transform the idea to written words. We greatly appreciate their efforts given the many constrains on their already busy 14-h work days.

It is the results and executive outcomes from our arduous days of toiling in the operating room and in the clinic chasing mastery that defines us. How our patients do and how well our trainees learn and feel mentored by us are the ultimate metrics. These predicates determine the culture we create and inculcate. It directly imparts patient experience and staff engagement. We are first processed focus not outcomes oriented. However, the ultimate metric of any continuous process improvement strategy are the results we yield. Nothing is more important than the patient's short-term and long-term outcomes we deliver. In this section we describe the newest and most unique measurement of outcomes and culture called the Efficiency Quality Index (EQI) (Cerfolio and Chang). It eliminates the three most common complaints doctors lament concerning administrative metrics: (1) the data is wrong (with EQI's the doctors validate their own data) (2) we measure the wrong metric (with the EQI the doctors or participant determine what are the metrics of quality for any procedure or operation) (3) data unfairly compares apples to oranges (with the EQI the staff members determine which patients or factors are included in the comparison, not administrators).

In addition, patient experience continues to be more commonly reported and important aspect of patient care—not just outcomes. Patient experience includes their interaction with our healthcare system, how well they can navigate their own electronic healthcare records, schedule tests, schedule blood work and communicate with our doctors, nurses and staff. These multiple touchpoints with our team and facilities defines their patient experience. In this special edition we describe how we have leveraged the innovative use of telemedicine (Ferrari-Light et al.) to improve our patient's experience over the past 10 years. This edition also describes in granular details how robotic surgery (Scheinerman et al.) and advanced bronchoscopic robotic skills (Jiang et al.) improve patients outcomes. Post-operative protocols further determine patients pain length of stay and experience (link —? Are they??). In our practice we now discharge the vast majority of patients to home within 23-h after robotic lobectomy and segmentectomy.

Finally, and perhaps the most important test of our legacy is how well we teach and mentor our trainees. Our mentorship maybe the most important aspect of our leadership. In this edition, we describe (Bhakhri et al.) the latest thoughts on simulation and when best to introduce it in our trainee's education. We also share thoughts and strategies concerning the importance of 3-D anatomy in pulmonary resection (Bhakhri et al.). Perhaps the most important aspect of surgical competency irrespective of your preferred operative platform: open thoracotomy, video-assisted or robotic, the master surgeon must know the anatomy from all vantage points. The surgeon must also know how to discern both common and unusually anatomic variants in order to avoid ligation of the inappropriate artery, vein or bronchus. I am honored to be associated with the many authors of this special edition and I greatly appreciate them taking the limited free time to write these articles and to educate us all.

## Author Contributions

The author confirms being the sole contributor of this work and has approved it for publication.

## Conflict of Interest

RC discloses no relationships over the last 2.5 years, but past relationships were with: C-SATS, ConMed, Fruit Street Health, Intuitive Surgical, ROLO-7, Tego.

## Publisher's Note

All claims expressed in this article are solely those of the authors and do not necessarily represent those of their affiliated organizations, or those of the publisher, the editors and the reviewers. Any product that may be evaluated in this article, or claim that may be made by its manufacturer, is not guaranteed or endorsed by the publisher.

